# 23 years of the discovery of *Helicobacter pylori*: Is the debate over?

**DOI:** 10.1186/1476-0711-4-17

**Published:** 2005-10-31

**Authors:** Niyaz Ahmed

**Affiliations:** 1 Pathogen Evolution Group and ISOGEM Collaborative Network on Genetics of Helicobacters (The International Society for Genomic and Evolutionary Microbiology), Centre for DNA Fingerprinting and Diagnostics, Hyderabad, India

## Abstract

The Gram negative curved bacillus *H. pylori *has become the prize bug of all times. Barry Marshall and Robin Warren the two discoverers of this organism have been awarded with this year's Nobel Prize. The Nobel committee at the Karolinska Institute of Sweden has selected this paradigm shift discovery of 1982 as the most impacting in medical sciences. This award has surprised many as the Nobel assembly has selected this 'Robert Koch styled medical detective work' for the prize as compared to many outstanding basic research stories on the waitlist. This editorial briefly touches the significant impact of *H. pylori *on gastroduodenal management and the path forward as the bug has become quite controversial in recent times.

*Helicobacter pylori *is a bacterium that colonizes human stomach and is an established cause of chronic superficial gastritis, chronic active gastritis, peptic ulcer disease and gastric adenocarcinoma [1]. The infection is on a fast decline in most of the western countries, mainly due to the success of therapeutic regimens and improved personal and community hygiene that prevents re-infection. The eradication in some of the countries has been quite promising and the pathogen was declared as an endangered bacterial species [2]. However, the situation is exactly opposite in many of the developing countries due to failure of treatment and emergence of drug resistance.

Barry J. Marshall and Robin Warren, two Australian researchers who discovered the bacterium *Helicobacter pylori *and deciphered its role in gastritis and peptic ulcer disease, have been awarded this year's Nobel Prize in Physiology or Medicine. The Nobel Assembly at the Karolinska Institute has honored them for their unexpected but paradigm shift discovery [3,4] that revealed that gastritis, and ulceration of the stomach or duodenum, were the result of infection with some curved Gram negative bacilli.

At that time when Warren and Marshall announced their findings, it was a long-standing belief in medical teaching and practice that stress and lifestyle factors were the major causes of peptic ulcer disease. Warren and Marshall rebutted that dogma, and it was soon clear that *H. pylori*, causes more than 90% of duodenal ulcers and up to 80% of gastric ulcers. The clinical community, however, met their findings, with skepticism and a lot of criticism and that's why it took quite a remarkable length of time for their discovery to become widely accepted. They had to just push it harder and harder with all experimental and clinical evidences. In 1985, for example, Marshall underwent gastric biopsy to put evidence that he didn't carry the bacterium, then deliberately infected himself to show that it in fact caused acute gastric illness. This 'self-help' experiment was published in the Medical Journal of Australia [4] to describe development of a mild illness over a course of 2 weeks, which included histologically proven gastritis. This extraordinary act of Marshall demonstrated extreme dedication and commitment to his research that generated one of the most radical and important impacts on the last 50 year's perception of gastroduodenal pathology. Their research made *H. pylori *infection one of the best-studied paradigms of pathogen biology, paving way for intense and hectic basic and clinical research activity leading to about 25,000 scientific publications till date. To realize the tremendous response of scientific and clinical communities, a dedicated journal called 'Helicobacter' was also started.

Soon the genomes of the bacilli were fully sequenced to decipher pathogenic mechanisms. Post genomic analyses have revealed interesting attributes of *H. pylori *genomic diversity, pathogenicity and novel mechanisms of causation of ulcer disease and cancer [5]. Efforts to know the implications of the genetic diversity of this bacterium have led to some interesting discoveries relating to its co-evolution with the human host, microevolution during infection and quasi-species development. Possible symbiotic relationships were debated since the discovery of this pathogen. However, the debate has been intensified since few years as some studies have posed the possibility that *H. pylori *infection may be beneficial in some humans [6-8]. Studies [8] have suggested that *H. pylori *infection protects against gastro-oesophageal reflux [8] and oesophageal carcinoma [9].

How long humans carried *H. pylori *is still a debatable issue. However, it is accepted that this organism has colonized humans possibly for many thousands of years, and the successful persistence of *H. pylori *in human stomach for such a long period may be a case to conceive that this organism is advantageous to its host.

It has been shown that *H. pylori *produces a cecropin-like peptide (antibacterial peptide) with high antimicrobial properties [6]. Another study [7] revealed that children infected with *H. pylori *were less likely to have diarrhoea than children without an infection, implying that *H. pylori *may be beneficial to human hosts. Interestingly, there has been a marked decline in the instances of peptic ulcer disease and gastric cancer in the 20^th ^century. Concurrent with this is a dramatic increase in the incidences of gastro-oesophageal reflux disease (GERD), Barrett's oesophagus and adenocarcinoma of the oesophagus in western countries [8,9]. This observation led to the speculation that *H. pylori *may in some way be associated with these diseases and perhaps capable of preventing their onset. Studies have also shown that *cagA*^+ ^*H. pylori *strains have a more protective effect than *cagA *^- ^strains [10]. The presence of *cagA*^+ ^*H. pylori *strains can reduce the acidity of the stomach, and it is believed that the raising of the pH by *H. pylori *prevents GERD, Barrett's oesophagus and adenocarcinoma of the oesophagus. Conversely, arguments have been made that, although *H. pylori *may prevent these reflux-associated diseases, the risks of acquiring gastric cancer via *H. pylori *infection far outweigh any possible benefits it may provide [11]. In spite of this controversy, recent reports have demonstrated a protective role for *H. pylori *in erosive reflux oesophagitis [11,12]. However, as safe and potent anitsecretory drugs are available it seems foolish to use a dangerous organism that has been associated with extremely dangerous outcomes such as a carcinoma.

In my opinion, the intricacies of the role of *H. pylori *in health and disease may be fully known only if we analyze pathogen biology as juxtaposed to the host biology and the environment (food and dietary habits). Cancer of stomach is a highly lethal disease and establishment of *H. pylori *as a risk factor for this malignancy deserves an approach to identify persons at increased risk; however, infection with this organism is extremely common and most colonized persons never develop cancer. The research activities should focus this reality, and identify populations that never develop gastric cancers despite heavy infection rates. Indians for example rarely develop gastric cancer; the incidence is extremely low or negligible (the age adjusted rates being extremely low, about 3 per 100, 000) [13] as compared to populations such as Japanese. This brings into debate a third dimension that is environment (diet? and lifestyle) that has not been explored as intensely as the pathogen virulence factors.

While many researchers are now convinced that the pathological outcomes of *H. pylori *infection are far more damaging than any beneficial effects of its inhabitation, there are enough strong evidences that absence of *H. pylori *from the stomach may lead to cancers of other gut regions. Therefore, there is an urgent need to thoroughly assess risks and benefits of *H. pylori *and the role of chronic infection in the development of cancers of gut and to provide for a basis to launch global strategies to fight this problem and settle the debate.
